# Conservative treatment versus surgical reconstruction for ACL rupture: A systemic review

**DOI:** 10.1016/j.jor.2024.05.026

**Published:** 2024-05-31

**Authors:** Zhongyu Jia, Johannes Greven, Frank Hildebrand, Philipp Kobbe, Jörg Eschweiler

**Affiliations:** a Department for Orthopaedic, Trauma and Reconstructive Surgery, RWTH Aachen University Hospital, Germany; b Department for Thoracic Surgery, RWTH Aachen University Hospital, Germany; c Department for Trauma and Reconstructive Surgery, BG Hospital Bergmannstrost Halle, Halle (Saale), Germany; d Department for Trauma and Reconstructive Surgery, University Hospital of the Martin Luther University Halle, Halle (Saale), Germany

**Keywords:** Knee, ACL rupture, Reconstruction, Conservative

## Abstract

**Background:**

Anterior cruciate ligament (ACL) rupture is a prevalent sports injury with rising rates attributed to increased population participation in sports activities. ACL rupture can lead to severe knee complications including cartilage damage, torn meniscus, and osteoarthritis. Current treatment options include conservative measures and surgical interventions. However, debates persist regarding the optimal approach.

**Purpose:**

This analysis intended to compare the function, knee stability, and incidence rate of secondary surgery between conservative and surgical treatments in ACL rupture patients.

**Methods:**

A systematic search was performed via Embase, Ovid Medline, PubMed, Cochrane Library, Web of Science, and Google Scholar for reporting outcomes of conservative and surgical treatments after ACL rupture. The outcomes included patient-reported outcome measures (PROMs), knee stability, the need for secondary meniscal surgery, delayed ACL reconstruction surgery, and revision ACL reconstruction surgery. Outcomes were analyzed using mean differences or odd ratios (OR) with 95 % CIs.

**Results:**

11 studies were included with 1516 patients. For PROMs, our evidence indicated no differences in KOOS Pain, KOOS Symptoms, KOOS Sport/Rec, KOOS ADL, and KOOS QOL. (all p > 0.05). for knee stability, pivot shift (OR, 0.14; p < 0.001), Lachman test (OR, 0.06; p < 0.001), and tibia translation (p < 0.001) were evaluated, and the available evidence favored surgical treatment over conservative treatment. For the incidence rate of any secondary surgery after the first diagnosis, the surgical group showed a lower rate of meniscal surgery with statistical significance (OR, 0.37; p < 0.001). The average rate of revision ACL reconstruction is 5.80 %, while the rate of delayed ACL reconstruction after conservative treatment is 18.51 %.

**Conclusion:**

Currently, there is insufficient empirical evidence to advocate a systematic surgical reconstruction for any patient who tore his ACL. This review found no differences in function outcomes between conservative and surgical treatments. Regarding knee stability and secondary meniscal surgery, the results prefer the surgical treatments. The occurrence rate of revision and delayed ACL reconstruction are non-negligible factors that must be fully understood by both surgeons and patients before choosing a suitable treatment.

## 1 Introduction

The incidence of anterior cruciate ligament (ACL) rupture, a prevalent sports injury, stands at approximately 46 injuries per 100,000 individuals annually in Germany.^1^ ACL ruptures happen especially in activities involving pivoting and sudden changes in direction. Moreover, the incidence rate has shown an upward trend in recent years. A widely accepted reason for this is the rising proportion of the population participating in activities and advancements in medical diagnostic technology.^2^^,^^3^

If the ACL rupture is not treated correctly, it can lead to further knee complications, such as cartilage damage, torn meniscus, and osteoarthritis.^4^, ^5^, ^6^, ^7^, ^8^, ^9^, ^10^ Currently, the two widely accepted treatments for ACL rupture are conservative and surgical.^11^, ^12^, ^13^ Conservative treatment refers to a series of therapeutic measures, directed by rehabilitation physicians, to restore knee strength, flexibility, and range of motion (ROM). However, it does not involve surgical intervention to restore the original anatomical structure of the knee joint; Surgical treatment predominantly involves the reconstruction or repair of the ligament, often utilizing a graft from patients such as the patellar tendon, quadriceps tendon, or hamstring tendon.^14^^,^^15^ However, some studies indicate a similar or higher rate of osteoarthritis (OA) from patients who underwent surgeries compared with conservative treatments.^16^, ^17^, ^18^, ^19^, ^20^, ^21^ Despite the preference for surgical intervention among patients and surgeons, the optimal treatment approach for ACL rupture is still debated.^22^^,^^23^ However, the determination between conservative therapy and surgical intervention should be formulated through a comprehensive assessment of individual patient factors, encompassing the gravity of the injury, functional objectives, and concurrent injuries.

In the past few years, most meta-analyses have encompassed studies on both open and arthroscopic surgeries.^13^^,^^16^^,^^24^^,^^25^ However, with the advancement of arthroscopic techniques, open surgical treatment for cruciate ligament injuries has become increasingly rare.^26^^,^^27^ Furthermore, existing meta-analyses have largely omitted studies on revision ACL reconstruction and meniscal repair surgeries.^13^^,^^16^^,^^25^^,^^28^^,^^29^

With this in mind, our research aimed to conduct a systematic review of the available evidence to compare conservative and surgical treatment in patients who had suffered an ACL rupture and had at least 12 months of follow-up (open surgeries are excluded). Specifically, we aimed to evaluate the effectiveness and potential risks of these treatments regarding patient-reported outcome measures (PROMs), knee stability, the incidence rates for secondary meniscal surgery, delayed ACL reconstruction surgery, and revision ACL reconstruction surgery.

## 2 Materials and methods

### 2.1 Search strategy

A literature search was conducted in December 2023 using the Preferred Reporting Items for Systematic Reviews (PRISMA) guidelines in six databases: Embase, Ovid Medline, PubMed, and Cochrane Library, Web of Science, and Google Scholar.^30^^,^^31^ The following keywords were used in the search process: (Anterior cruciate ligament OR ACL) AND ((reconstruction OR repair OR operation OR surgery) AND (non-surgical OR conservative OR non-operation OR rehabilitation OR physiotherapy OR brace)).

Conservative treatment was defined as treatment that did not include any surgical reconstruction. The following aspects of conservative treatment were extracted: bracing, weight-bearing restrictions, range-of-motion restrictions, physiotherapy, strength, plyometrics, etc. Surgical treatment includes only arthroscopy for ACL repair or reconstruction, and there was no restriction regarding the source of the tendon if needed.

The articles for our study were limited to those written in English.^32^ Two reviewers (Z. J, J. G) reviewed all studies for titles and abstracts independently. Then, full texts for inclusion with the criteria below, and the disagreement was solved by consensus or by the intervention of a third author (J. E).

The inclusion and exclusion criteria for our study are shown in Table 1.

**Table 1:** Inclusion and exclusion criteria for study selection.

Inclusion Criteria	Exclusion Criteria
• Surgical and conservative treatment groups for ACL injuries • Original scientific research articles	• Revision of ACL reconstruction • Open reconstruction • Case reports or small case series (any group <10 patients) • A follow-up period of less than 12 months • Non-comparative studies • Articles written in a language other than English • Nonclinical studies (e.g. biomechanical, cadaveric, or animal models)

### 2.2 Methodological quality of studies

The evaluation of methodological quality was conducted utilizing “Modified Coleman Methodology Score” (mCMS) as employed in prior literature.^33^^,^^34^ The mCMS estimation is derived from ten distinct dimensions: study size, mean follow-up, surgical approach, type of study, description of diagnosis, surgical technique, postoperative rehabilitation, outcome criteria, procedure of assessing outcomes, and participant selection process. Every dimension has an individual subscore that contributes to an aggregate score, with a maximum potential value of 100. The mean composite scores, together with their corresponding standard deviations, were subsequently computed.

### 2.3 Data extraction

We collected the data in Excel 2016 (Microsoft Corp.) first. The data comprised the literature detail, patient characteristics, experience data, and incidence of secondary surgery. The literature detail included the author's names, year of publication, number of patients, DOI, study design, and remarks. Patient characteristics included the age (surgery time), gender, and the follow-up period. The experience data included the PROMs, knee laxity data, and incidence of subsequent knee surgery. KOOS pain, KOOS symptoms, function in sport and recreation (KOOS Sport/Rec), function in daily living (KOOS ADL), and knee-related quality of life (KOOS QOL) were collected as PROMs. Lachman test, pivot shift test, and tibia translation (side-to-side difference) measured by KT-1000 arthrometer were classified as knee laxity. Secondary meniscal surgery includes meniscal repairs, manipulation, total or partial meniscectomy, etc., delayed reconstruction surgeries after conservative treatment, and revision reconstruction surgeries after surgical treatment including all kinds of operative approaches. The secondary knee surgeries are calculated during the follow-up period after the first diagnosis. If mean value and standard deviations (SD) were unavailable, they were calculated based on the previously defined methods.^35^

### 2.4 Synthesis methods

The primary author (Z. J) conducted statistical analyses adhering to the Cochrane Handbook for Systematic Reviews of Interventions. The p-value <0.05 was considered statistically significant. For the systemic review, the software Review Manager version 5.4 (The Nordic Cochrane Collaboration, Copenhagen) was used. Continuous variables were analyzed using the mean value and standard deviation (SD), while binary variables were assessed using the odds ratio (OR) effect measure. A 95 % confidence interval (CI) was employed for all comparisons. Heterogeneity was estimated using the Higgins-I^2^ tests, with values < 50 % and >50 % indicating low and high heterogeneity, respectively. For low heterogeneity, a fixed model effect was applied; a random model effect was applied for high heterogeneity. The Egger's test and the degree of asymmetry observed in the funnel plot were employed to assess and illustrate the potential risk of publication bias, respectively.

## 3 Results

### 3.1 Study selection and characteristics

**Fig. 1:**
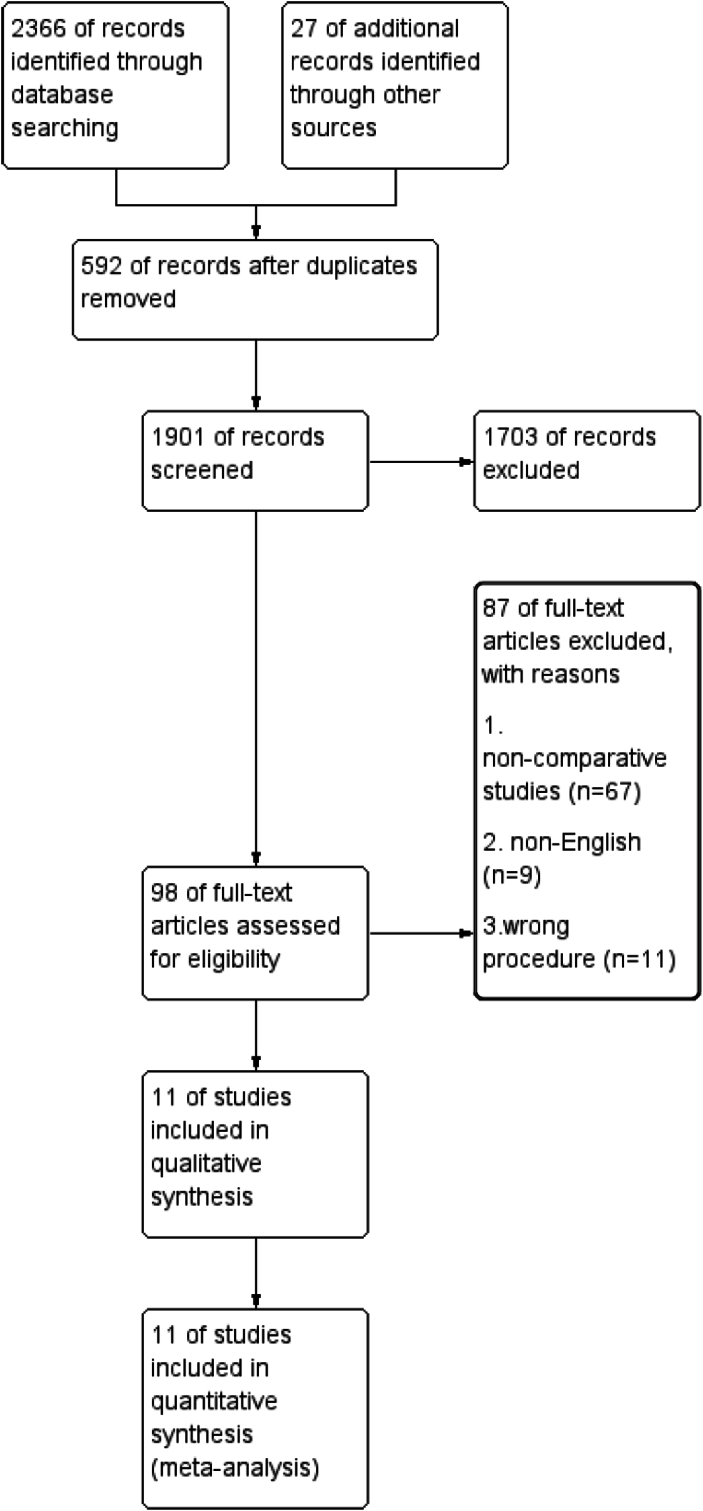
Flow diagram of study selection.

In total, 2393 articles were initially identified. The flowchart of the literature selection process based on PRISMA is shown in Fig. 1. 592 articles were excluded first because of duplicates, and 1703 articles were excluded because of the eligibility based on title and abstract. An additional 87 articles were excluded due to non-comparative study designs, non-English language, and incorrect procedures. Finally, a total of eleven articles were included in the final quantitative analysis.

Of the eleven articles, six of them were prospective cohort studies including two randomized controlled trials. The other five articles were retrospective studies. Three studies had part of the data that satisfied inclusion and exclusion criteria, while other data failed to satisfy these criteria. In these instances, only patients satisfying our inclusion criteria were included in the quantitative analyses. Overall, 1516 patients were included in this analysis, 895 in the surgical treatment group, and 621 in the conservative treatment group. The mean number of patients per study was 134.90 ± 98.78 (range, 32–380).

The presence of meniscal injury at initial diagnosis was reported in three studies, and three studies reported injuries to ligaments other than the ACL. For surgical treatment, all the interventions were performed by orthopedic surgeons specializing in knee surgery.

For graft choice, five studies chose the bone-patellar tendon-bone autograft and one study chose the semitendinosus tendon autograft, three studies chose more than one type of graft, two studies did not report the type of graft. For conservative treatment, all conservative treatments in the studies included a progressive physical therapy program led by experienced physical therapists. Three studies mentioned the knee brace during the rehabilitation program. For follow-up period, three studies had a follow-up period over one year but less than or equal to three years, three studies had over three years but less than or equal to five years, two studies had over five years but less than or equal to ten years, and three studies had over ten years follow-up period.

The summary of the included 11 articles are shown in Table 2.

**Table 2:** Study generalities and patient demographic.

Author	Year	Study Design	Follow-Up (mean months)	Patients(n)	Mean Age(y)	Female (%)
S. P. Zysk	2000	retrospective		133	46	50
C. Fink	2001	retrospective	156	71	C^a^: 27.9	C: 28
					S^b^:33.6	S: 20
D. Fithian	2005	prospective	79	209	C: 40.0	C: 54
					S: 36.3	S: 38
A. Meunier	2007	prospective		68	C: 21	C: 38
					S: 22	S: 25
D. E. Meuffels	2009	retrospective	120	50	C: 37.8	C: 24
					S: 37.6	S: 24
N. Streich	2011	retrospective	180	80	C: 24.0	C: 30
					S: 26.0	S: 30
R. Frobell	2013	RCT^c^	60	120	C: 25.8	C: 34
					S: 26.4	S: 20
D. Yperen	2018	retrospective	240	50	C: 27.8	C: 24
					S: 27.6	S: 24
E. Wellsandt	2018	prospective		107	C: 31.8	C: 55
					S: 28.6	S: 33
M. Ehlinger	2021	prospective		380	C: 59.9	C: 62
					S: 54.8	S: 59
D. Beard	2022	RCT		248	C: 32.9	C: 38
					S: 32.9	S: 29

a Conservative treatment.

b Surgical reconstruction.

c Randomized controlled trial.

### 3.2 Quality of evidence

The methodological quality was evaluated using the modified Coleman Methodology Score (mCMS). The findings indicate a predominantly moderate ranking across the studies, with individual scores ranging from 56 to 79. The mean mCMS value among the 11 studies was 67.6, with a standard deviation of 10.9. One study was categorized as "poor," while 10 were deemed "fair." Significant methodological shortcomings were identified in the areas of diagnostic description, surgical approach delineation, detailing of surgical techniques, and descriptions of postoperative rehabilitation.

### 3.3 Outcomes

#### 3.3.1 PROMs

Similarity was found in KOOS Pain (p = 0.25), KOOS Symptoms (p = 0.43), KOOS Sport/Rec (p = 0.90), KOOS ADL (p = 0.08), and KOOS QOL (p = 0.58).

#### 3.3.2 Joint stability

The surgical group showed lower rates of the Lachman test (OR 0.06; p < 0.0001), positive pivot shift (OR 0.14; p = 0.0006), and less tibia translation (p < 0.00001).

#### 3.3.3 Incidence of secondary meniscal surgery

**Fig. 2:**
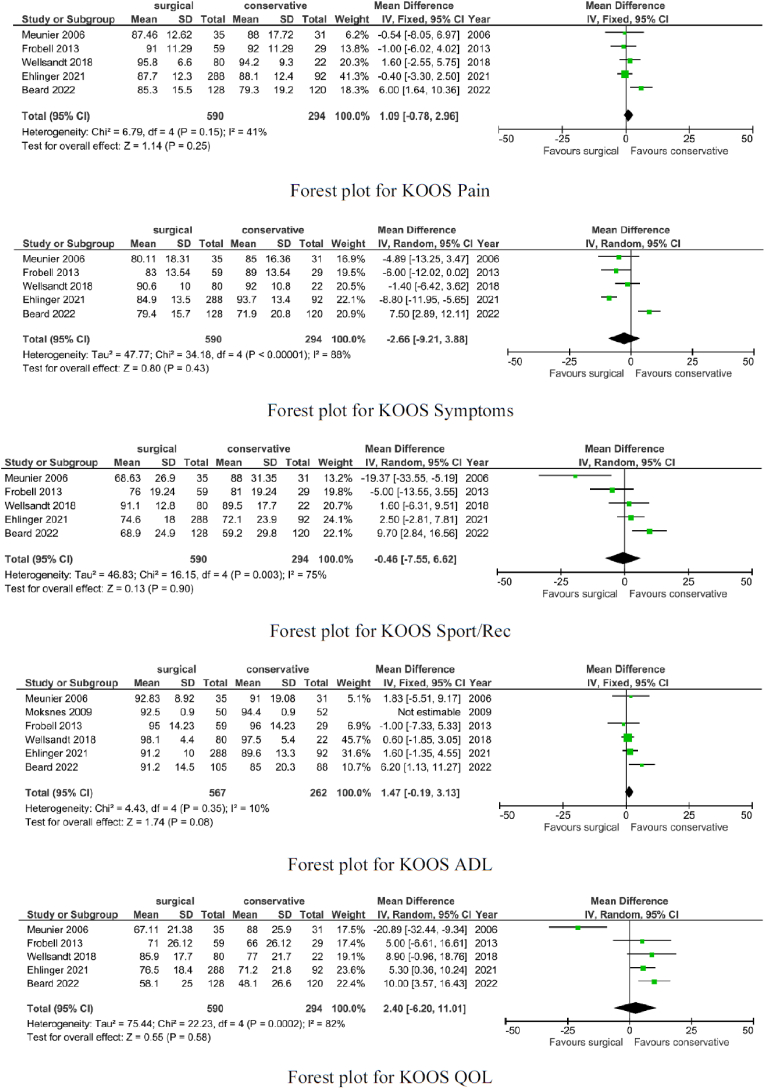
Forest plots for PROMs scores.

**Fig. 3:**
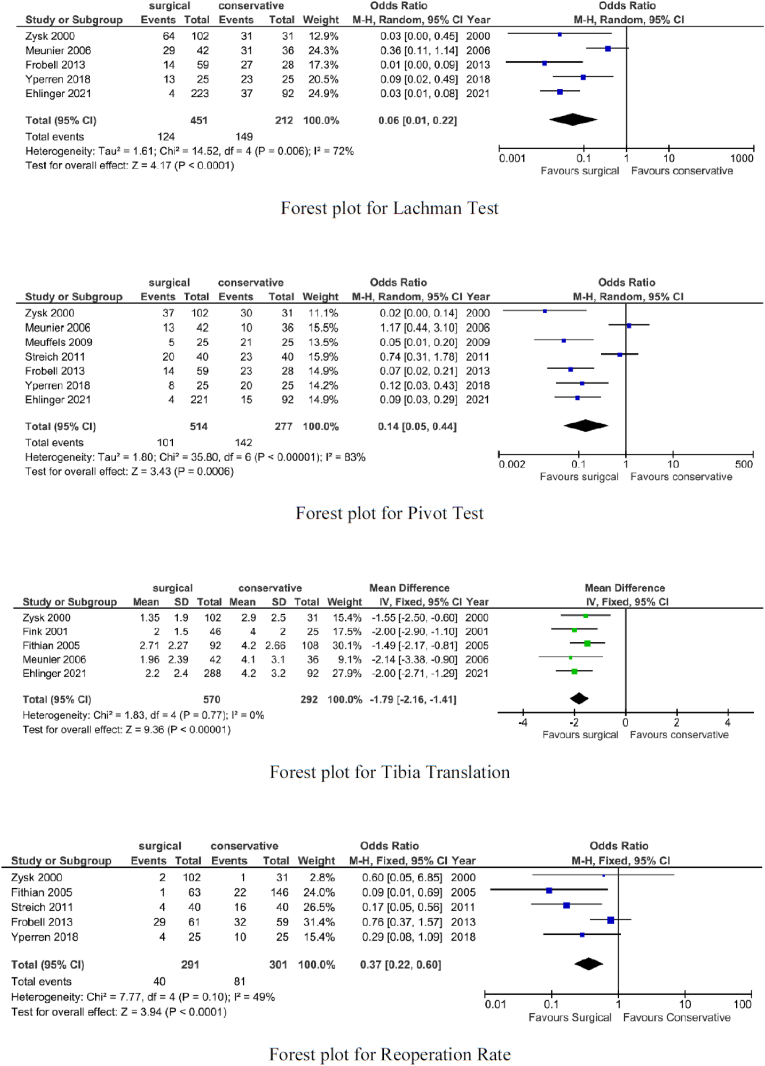
Forest plots for knee laxity and operation rate.

The surgical group showed lower rates of incidence of secondary meniscal surgery (OR 0.37; p < 0.0001). These results with more details are shown in Fig. 2, Fig. 3.

#### 3.3.4 Incidence of delayed reconstruction and revision ACL reconstruction surgery

Only four articles mentioned the revision ACL reconstruction surgery, the incidence rates are 4.76 % (1/21), 7.14 % (3/42), 3.28 % (2/61), and 8 % (2/25), respectively, and the average amount of rate is 5.80 %. seven7 articles mentioned the delayed reconstruction ACL surgery after conservative treatments, the incidence rates are 3.22 % (1/31), 8 % (2/25), 22.58 % (7/31), 30.77 % (16/52), 10.17 % (6/59), 50.85 % (30/59), 4 % (1/25), respectively, the average amount of rates is 18.51 %.

#### 3.3.5 Summary

When the knee function outcomes were compared between surgical and conservative treatments. All other outcomes were similar among KOOS Pain, KOOS Symptoms, KOOS Sport/Rec, KOOS ADL, and KOOS QOL. When risk of the knee stability: pivot shift, Lachman test, and tibia translation were evaluated, the available evidence strongly favored the surgical treatment over conservative treatment. For the incidence rate of any secondary surgery after the first diagnosis, the surgical group showed a lower rate of meniscal surgery. The mean revision rate for ACL reconstruction is 5.80 %, whereas the rate of delayed ACL reconstruction following conservative treatment is 18.51 %.

### 3.4 Publication bias

**Fig. 4:**
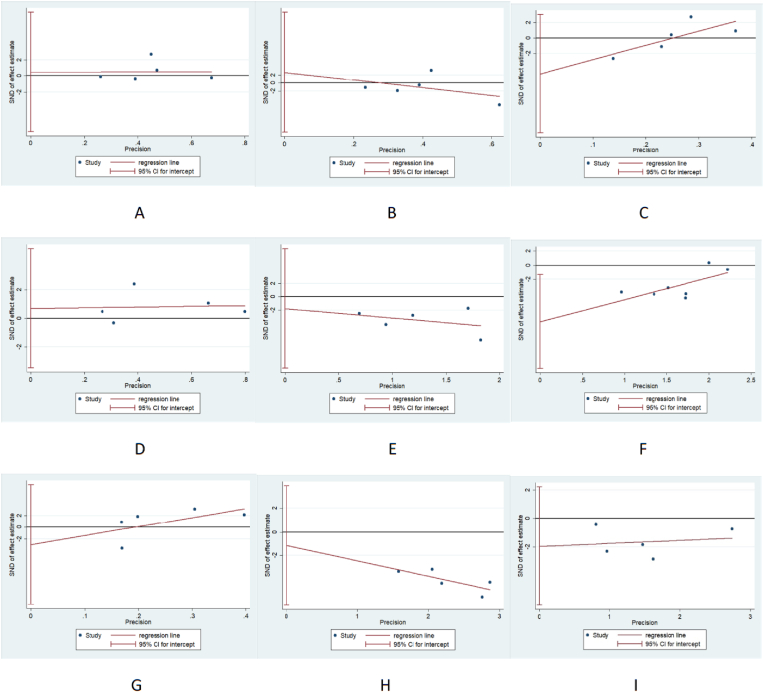
p-values of Egger's test and funnel plots, A: KOOS Pain; B: KOOS Symptoms; C: KOOS Sport/Rec; D:KOOS ADL; E:KOOS QOL; F:Lachman test; G:Pivot shift test; H:Tibia translation; I:Reoperation rate.

No evident asymmetrical pattern was observed in the funnel plots, indicating a low risk of publication bias. The funnel plots and p-values from Egger's test for this systematic review are depicted in Fig. 4.

## 4 Discussion

Our study found that the surgical treatments for ACL rupture have similar PROMs results, but less laxity compared to the conservative treatment. Moreover, patients in the conservative treatment group were more likely to undergo secondary meniscal surgery in the future.

An increasing number of surgeons have expressed a preference for surgical intervention in managing ACL ruptures. One of the aims of such an intervention is to restore the function of ACL, particularly in terms of constraining anterior tibial translation and internal rotation 36,37. Our research findings found that patients who underwent surgical treatment exhibited similar PROMs data when compared with those who received conservative treatment. This is in contradiction with a recent randomized controlled trial on the reconstruction versus conservative treatment of non-acute ACL injuries 38. The trial suggested that immediate reconstruction for symptomatic non-acute ACL rupture could yield better PROMs. We believe the main reason for this is that the follow-up period of this randomized controlled trial (RCT) was only 18 months, and there is evidence that establishing new neuromuscular control takes time 34,39, 40, 41, 42. As the follow-up period extended, we saw functional scores gradually recovering. An earlier RCT study with a longer follow-up period also confirmed our point 43. There was no significant difference in PROMs scores between the conservative treatment of ACL rupture and the surgical group in follow-ups up to five years after injury. In addition, the article also mentioned that for patients in the conservative treatment group who initially did not choose surgery but later underwent surgery due to persistent symptoms, there was no statistically significant difference in returning to pre-injury sports levels after the injury 38. The reason here may be caused by the change in patients' lifestyles and the reduction of expectations for knee joint function 44, 45, 46. Therefore, reconstruction is not the only option for recovering the function of the knee joint.

An increasing number of scholars advocate for early surgical treatment of ACL rupture among young patients 46, 47, 48, 49. As these patients are likely to continue participating in knee rotational sports in the future, early reconstruction aids in restoring knee joint stability and minimizing the risk of secondary meniscus injury 50, 51, 52. Our research results corroborate this perspective. However, despite the benefits of reconstruction surgery in terms of knee joint stability and reduced risk of secondary meniscus injury during the follow-up period, a growing number of studies indicate no significant long-term difference between surgical and conservative treatment methods concerning knee joint function, activity levels, and radiographic osteoarthritis changes 53, 54, 55, 56, 57. Notably, some studies demonstrate that even highly active ACL patients who have received conservative treatment achieve similar subjective and functional outcomes to those who underwent surgery over a long-term period 17,55,58. Consequently, while knee stability can be addressed by reconstruction, it may not necessarily dictate future outcomes.

With the advancement of arthroscopic technology, open surgeries have gradually become obsolete, and arthroscopic surgical treatment for ACL injuries has become the preferred surgical approach for most physicians 1,59,60. As a result, unlike most literature that included both open and arthroscopic surgeries for the treatment of ACL injuries 13,16,24,25, our research focused exclusively on arthroscopic interventions. Additionally, our study addressed a commonly overlooked aspect in many current meta-analyses, which is the variation in secondary surgery rate under different treatments 13,16,25,28,29. Providing comprehensive information to patients about potential outcomes not only upholds their right to informed consent but also reduces medical-patient conflicts arising from information asymmetry. Consequently, our systemic review excluded studies involving open surgeries for the treatment of ACL injuries. Furthermore, our research specifically focused on investigating the reoperation rates associated with different treatment approaches. This systemic review is expected to assist physicians in thoroughly explaining potential possibilities to patients, thereby fully safeguarding patient rights.

## 5 Limitations

This systemic review also has some limitations. Firstly, our research includes randomized and non-randomized studies, which lowers the overall level of evidence. Secondly, some prospective experiments lacked blinding in their design. Especially in terms of how to group the surgical and conservative treatment groups, in addition, some patients with combined injuries, are often directly classified into the surgical group, which increases the risk of selection bias. Thirdly, our research is limited by different patient ages, research designs, and follow-up lengths, especially patient age distribution, choices of grafts, designs of conservative treatment rehabilitation programs, and different follow-up cycles and nodes. These variations make direct comparisons difficult and could potentially lead to overestimation or underestimation of conclusions. Lastly, as with all systematic reviews, our search strategy may have missed some relevant studies.

## 6 Conclusion

There is insufficient evidence to advocate an optimal treatment for any patient who tore his ACL. For relatively young patients with ACL rupture, they can be suggested to take ACL reconstruction, because reconstruction can restore stability and reduce current symptoms of knee joint. Certainly, surgeons should also inform patients that no matter which treatment is chosen, future active and passive knee joint function, and restoration of pre-injury sports abilities, may not have a significant difference. However, these patients who choose the conservative treatments are more likely to undergo a second surgery. Therefore, the clinicians should give the choice of treatment to the patient's hand while informing the patient of the pros and cons in the short and long term.

## Patients consent

None.

## Ethical statement

None.

## Funding

Zhongyu Jia was supported by the 10.13039/501100004543China Scholarship Council.

## CRediT authorship contribution statement

**Zhongyu Jia:** Conceptualization, Methodology, Investigation, Writing – original draft. **Johannes Greven:** Data curation, Writing – review & editing. **Frank Hildebrand:** Writing – review & editing, Supervision. **Philipp Kobbe:** Writing – review & editing. **Jörg Eschweiler:** Data curation, Writing – review & editing, Supervision.

## Declaration of competing interest

None.
